# Association of *MMP - 12 *polymorphisms with severe and very severe COPD: A case control study of *MMP*s - *1, 9 and 12 *in a European population

**DOI:** 10.1186/1471-2350-11-7

**Published:** 2010-01-15

**Authors:** Imran Haq, Sally Chappell, Simon R Johnson, Juzer Lotya, Leslie Daly, Kevin Morgan, Tamar Guetta-Baranes, Josep Roca, Roberto Rabinovich, Ann B Millar, Seamas C Donnelly, Vera Keatings, William MacNee, Jan Stolk, Pieter S Hiemstra, Massimo Miniati, Simonetta Monti, Clare M O'Connor, Noor Kalsheker

**Affiliations:** 1 School of Molecular Medical Sciences, Institute of Genetics, Queen's Medical Centre, University of Nottingham, UK; 2 Therapeutics and Molecular Medicine, Queen's Medical Centre, University of Nottingham, UK; 3 UCD School of Public Health and Population Science, University College Dublin, Belfield, Dublin, Ireland; 4 Pulmonary Dept, CIBERES, Hospital Clinic, Hospital Clínico y Provincial de Barcelona, Villarroel, Barcelona, Spain; 5 Lung Research Group, Dept of Clinical Science at North Bristol, Southmead Hospital, University of Bristol, Westbury on Trym, Bristol, UK; 6 UCD School of Medicine and Medical Science, UCD Conway Institute, University College Dublin, Ireland; 7 Letterkenny General Hospital, Letterkenny, Donegal, Ireland; 8 Respiratory Medicine, ELEGI Colt Laboratories, Wilkie Building, University of Edinburgh, Edinburgh, UK; 9 Dept of Pulmonology (C3-P), Leiden University Medical Center, Leiden, The Netherlands; 10 Department of Medical and Surgical Critical Care, University of Florence, Italy; 11 CNR Institute of Clinical Physiology, Pisa, Italy

## Abstract

**Background:**

Genetic factors play a role in chronic obstructive pulmonary disease (COPD) but are poorly understood. A number of candidate genes have been proposed on the basis of the pathogenesis of COPD. These include the *matrix metalloproteinase *(*MMP*) genes which play a role in tissue remodelling and fit in with the protease - antiprotease imbalance theory for the cause of COPD. Previous genetic studies of *MMP*s in COPD have had inadequate coverage of the genes, and have reported conflicting associations of both single nucleotide polymorphisms (SNPs) and SNP haplotypes, plausibly due to under-powered studies.

**Methods:**

To address these issues we genotyped 26 SNPs, providing comprehensive coverage of reported SNP variation, in *MMP*s- 1, 9 and 12 from 977 COPD patients and 876 non-diseased smokers of European descent and evaluated their association with disease singly and in haplotype combinations. We used logistic regression to adjust for age, gender, centre and smoking history.

**Results:**

Haplotypes of two SNPs in *MMP*-12 (rs652438 and rs2276109), showed an association with severe/very severe disease, corresponding to GOLD Stages III and IV.

**Conclusions:**

Those with the common A-A haplotype for these two SNPs were at greater risk of developing severe/very severe disease (p = 0.0039) while possession of the minor G variants at either SNP locus had a protective effect (adjusted odds ratio of 0.76; 95% CI 0.61 - 0.94). The A-A haplotype was also associated with significantly lower predicted FEV_1 _(42.62% versus 44.79%; p = 0.0129). This implicates haplotypes of *MMP-12 *as modifiers of disease severity.

## Background

Chronic Obstructive Pulmonary Disease (COPD) is a major cause of morbidity and mortality worldwide. The World Health Organisation's Global Burden of Disease and Risk Factors project estimates that in 2001, COPD was the fifth leading cause of death in high-income countries and the sixth leading cause of death in countries of low and middle income [1]. Cigarette smoking is the main aetiological risk factor in disease development. However, only about 10 - 20% of smokers develop clinically significant COPD though this may be an under-estimate [2]. Furthermore, there is variability between smokers who have similar levels of smoke exposure, with many developing milder forms of the disease [3]. These observations suggest that underlying genetic factors contribute to either disease susceptibility or protection.

A widely accepted hypothesis of COPD causation is an imbalance of proteolytic enzymes and their inhibitors based on the observation that severe alpha_1_-antitrypsin deficiency predisposes cigarette smokers to the development of pulmonary emphysema in early adult life. This is thought to be due to uninhibited neutrophil elastase (NE) activity degrading elastin, a major component of lung connective tissue [4]. There are a number of other proteolytic enzymes also capable of degrading elastin, most particularly the *matrix metalloproteinase*s (*MMP*s), a family of potent proteinases that degrade all the major protein components of the extra-cellular matrix (ECM).

*MMP*s play a key role in tissue remodelling and repair and there is significant evidence that members of the *MMP *family may also play an important role in COPD pathology. Transgenic mice over-expressing *MMP-1 *develop emphysema [5], whilst *MMP-12 *knockout mice are protected from emphysema despite prolonged cigarette smoke exposure[6], implicating *MMP-12 *as a compelling emphysema determinant in this model. In COPD patients a range of studies implicate *MMP*s in the pathogenesis of the disease [7-12]. A number of studies have reported associations of genetic variants in *MMPs-1, 9 and 12 *with COPD or related phenotypes. *MMP-1 *and *MMP-12 *are located in close proximity on chromosome 11 and *MMP-9 *is located on chromosome 20. Reported associations of these genes with COPD include associations of haplotypes of *MMP*s- *1 and 12 *with rate of decline in lung function [13] in Caucasians and association of an *MMP-9 *promoter polymorphism with emphysema in a Japanese population [14]. However, there are conflicting data on the potential involvement of *MMP *variation in COPD. This likely reflects a number of issues relating to false positives in case- control studies or a lack of power due to relatively small sample size and the limited number of single nucleotide polymorphisms (SNPs) investigated, *MMP 1, 9 and 12 *studies are summarised in table 1. Other issues that contribute to lack of reproducibility include variation in the definition of cases and controls, improper matching of cases and controls and ethnic differences [15]. The results therefore need to be interpreted with caution.

**Table 1:** Previous association studies performed in COPD

Author (Year)	Case	Control	Population	Gene (Polymorphism)	rs number	Findings
Minesmatu *et al *(2001) [14]	45 COPD, emphysema on CT-Scan	65 Smokers, No emphysema on CT-Scan	Japanese	MMP9 (-1562C/T)	rs3918242	T allele with Emphysema DLco/VA lower and emphysemous changes distinct with C/T and T/T

Joos *et al *(2002) [13]	284 fast rate of lung function decline	306 slow rate of lung function decline	Caucasian (USA)	MMP1 (-1607G/GG) MMP9 (CA Repeat) MMP9 (-1562C/T) MMP12 (-82A/G) MMP12 (357Asn/Ser)	rs1799750 N/A rs3918242 rs2276109 rs652438	-1607GG associated with fast rate of decline of lung function Haplotypes of 357Asn/Ser and -1607G/GG associated with rate of decline of lung function

Zhou *et al *2004) [33]	100 COPD	98 healthy smokers	Han (South China)	MMP9 (-1562C/T)	rs3918242	C/T and T allele frequencies significantly higher in COPD patients

Zhang *et al* (2005) [30]	147 COPD	120 healthy smokers	Han (North China)	MMP1 (-1607G/GG) MMP9 (-1562C/T) MMP12 (-82A/G) MMP12 (357Asn/Ser)	rs1799750 rs3918242 rs2276109 rs652438	357 Asn/Asn is a risk factor for COPD

Ito *et al *2005) [34]	84 COPD	85 healthy smokers	Japanese	MMP9 (-1562C/T)	rs3918242	T allele associated with upper lung dominant emphysema

Hersh *et al *2006) [35]	304 COPD 127 families early onset COPD	441 healthy smokers N/A	Caucasian (USA)	MMP1 (-1607G/GG) MMP9 (CA Repeat)	rs1799750 N/A	No Association found

Tesfaigzi et al (2006) [25]	385 male COPD with > 20 Pack Years	N/A	Caucasian (USA)	MMP1 (-1607G/GG) MMP9 (279 R/Q) MMP9 (-1562C/T) MMP9 (CA Repeat)	rs1799750 rs17576 rs3918242 N/A	Homozygous 279Arg associated with 3× risk for COPD. Haplotype containing 279Arg and CA associated with COPD risk.

Schirmer *et al *(2009) [36]	111 COPD	101 Non smoking controls	Caucasian (Brazil)	MMP9 (-1562C/T) MMP12 (-82A/G)	rs3918242 rs2276109	No Association found

To address the major issues, a European cohort of 977 Caucasian COPD patients and 876 non-diseased smoking controls with complete genotyping information was used to evaluate the relationship of 26 SNPs within *MMP*s- *1, 9 *and *12 *with COPD and to investigate associations with GOLD -defined severity phenotypes. These tagging SNPs provide coverage of most of the variation within the *MMP *genes.

## Methods

### Subjects

COPD and non-diseased smoking control subjects were recruited from six European centres. Only white Caucasians were recruited. Study approval was obtained from appropriate local committees to each recruitment centre. Informed consent was obtained from all subjects. The resource of COPD patients and non-diseased smoking controls has been described previously [16-19] and is one of the largest reported to date. The number of subjects recruited from each centre was as follows. Barcelona (Spain): 70 controls and 138 cases; Bristol (UK): 152 controls and 129 cases; Dublin (Ireland): 195 controls and 196 cases; Edinburgh (UK): 81 controls and 168 cases; Leiden (the Netherlands): 216 controls and 188 cases; and Pisa (Italy): 198 controls and 198 cases. It should be noted that the total resource consisted of 1017 COPD patients and 912 controls. If strict criteria for genotyping were not met or assay design proved difficult, results were not included in the present analysis to minimise potential errors. This resulted in completed unequivocal genotypes in 977 COPD patients and 876 controls.

Briefly, recruitment criteria were agreed by a panel of respiratory physicians with an interest in COPD and included a firm clinical diagnosis of COPD; airflow limitation as indicated by (Forced Expiratory Volume in 1 Second) FEV_1 _≤ 70% normal predicted values and FEV_1_/(Forced Vital Capacity) FVC < 0.7; no significant reversibility with salbutamol (≥ 200 μg) and a smoking history of ≥ 20 pack years with matched smoking history for the controls. FEV_1 _is measured using a spirometer, it is the maximum volume of air that can be forcibly breathed out in one second after full inspiration. FVC is the maximum volume of air that can be forcibly breathed out after full inspiration.

Patients were excluded from the study if they had an established diagnosis of asthma, lung cancer, history of atopy, known AAT deficiency or a serum AAT level of less than 1.0 g/L. Patients with acute exacerbations four weeks preceding study assessment were also excluded. This was an agreed part of the protocol to enable future studies, where appropriate, to investigate correlations of genotypes with quantitative traits such as lung function or concentrations of analytes in biological fluids. We did not recruit any patients with Global Obstructive Lung Disease (GOLD) Stage I. Disease severity was classified according to GOLD as follows: GOLD Stage II (moderate disease): FEV_1_/FVC ratio of < 0.70 and FEV_1 _predicted ≥ 50% and < 80%; GOLD Stage III (severe disease): FEV_1_/FVC ratio of < 0.70 and FEV_1 _predicted ≥ 30% and < 50%; and GOLD Stage IV (very severe disease): FEV_1_/FVC ratio of < 0.70 and FEV_1 _predicted < 30% [20].

Control subjects with no evidence of airflow obstruction (FEV_1 _and FVC ≥ 80% and FEV_1_/FVC > 0.70) were recruited at each centre with attempted matching to COPD patients for ethnicity, age, gender and smoking history. Exclusion criteria were as described for cases and additionally included family history of COPD. However, complete matching was not achieved due to a high proportion of smokers aged 65 or over having evidence of some pulmonary obstruction, thereby excluding them from recruitment as controls.

We compared the frequencies of *MMP *SNPs for each centre in cases and controls to look for population stratification. For the genes under study, population stratification would have been reflected in different frequencies in the centre to centre comparisons. No such differences were observed. These findings are in keeping with previous observations on this sample set [19] and with data obtained from British samples reported by the Wellcome Trust Case Control Consortium in genome- wide association studies where stratification has been shown to be not as significant an issue in the British population as previously thought [21]; similar findings are likely for western European populations.

### Study Population Characteristics

The study population characteristics are summarised in Table 2. Despite attempts to match cases and controls there were significant differences observed and adjustments were made by logistic regression to take this into account in the statistical analysis. The characteristics of recruited COPD patients across the GOLD severity categories are shown in Table 3.

**Table 2:** Characteristics of Controls and COPD subjects

	Control	COPD	P-value
**Male (%)**	63.2	69.9	0.3554
**Age (yr)**	60.7 ± 8.9	65.9 ± 8.2	< 0.0001
**Smoking Pack Years**	38.6 ± 17.4	48.7 ± 22.8	< 0.0001
**Predicted FEV_1 _(%)**	95.4 ± 11.0	43.0 ± 15.1	< 0.0001
**FEV_1_/FVC (%)**	77.8 ± 4.9	47.4 ± 12.1	< 0.0001
**N**	876	977	

Values indicated are means ± SD. Pack Years missing for 6 persons.

**Table 3:** Characteristics of COPD patients according to GOLD classification of disease severity

GOLD Status	II Moderate	III Severe	IV Very Severe	P-value
**Male (%)**	74.7	67.5	67.2	0.0608
**Age (yr)**	66.1 ± 8.0	66.7 ± 8.4	64.3 ± 8.1	0.0016
**Pack Years**	49.2 ± 21.1	48.5 ± 22.3	48.4 ± 26.0	0.9105
**Predicted FEV_1 _(%)**	60.2 ± 5.9	40.0 ± 5.8	23.4 ± 4.3	< 0.0001
**FEV_1_/FVC (%)**	57.1 ± 7.8	45.9 ± 9.9	36.2 ± 9.4	< 0.0001
**N**	336	406	235	

Values indicated are means ± SD. Pack years missing for 3, 2, 1 persons per classification respectively.

### Power Calculations

Using Quanto software for the power calculations, with an autosomal dominant model, this study has 80% power to detect an odds ratio of 1.5 with an allele frequency of 0.1.

### SNP Selection and Haplotypes

SNP identification within *MMP*s- 1, 9 and 12 genes was carried out using information obtained from a combination of databases including HapMap and Seattle SNPs. SNPs with a minor allele frequency of less than 5% were excluded from the study, as these were likely to be insufficiently powered to detect association. SNPs lacking any validation status as described on the dbSNP database were also excluded. Further narrowing of SNP selection was based on putative function described in the databases and predicted function by the FastSNP web-service [22]. Where SNPs were in linkage disequilibrium (r^2 ^≥ 0.8) in previously reported Caucasian populations (HapMap, National Heart Lung and Blood Institute's (NHLBI) Programs for Genomic Applications European (PGA EUR) population, or Environmental Gene Project Centre d'Etude du Polymorphisme Humain (EGP CEPH) European population) a single tagging SNP was chosen for genotyping, thus minimising the number of SNPs that needed to be investigated and reducing the number of statistical tests required. This resulted in 26 SNPs across the three genes being selected for analysis. Information on the 26 genotyped SNPs is indicated in Table 4. The SNPs were selected as described below.

**Table 4:** SNPs selected for genotyping

**SNP No**.	Gene	Locus	tagged SNPs	Location in Gene	Contig position	Major/Minor allele
**1**	*MMP-1*	rs2071230	rs7125320	3' UTR	18098075	A/G
				Intronic	18095326	G/T
**2**	*MMP-1*	rs2239008	rs2071232	3' UTR	18097954	G/A
			rs996999	Intronic	18093365	T/C
				Intronic	18091971	C/T
**3**	*MMP-1*	rs470215	rs5854	3' UTR	18097935	A/G
			rs470747	3' UTR	18098160	C/T
			rs470132	Intronic	18097439	T/C
				Intronic	18092477	G/T
**4**	*MMP-1*	rs1938901	rs5031036	Intronic	18097369	C/T
**5**	*MMP-1*	rs17881293		Intronic	18096568	A/G
**6**	*MMP-1*	rs17884110		Intronic	18095557:18095558	-/C
**7**	*MMP-1*	rs17878931		Intronic	18092379	C/A
**8**	*MMP-1*	rs10488		Exonic	18091012	G/A
				Intronic	18092870	A/G
**9**	*MMP-1*	rs470358	rs505770	Intronic	18090333	C/T
**10**	*MMP-1*	rs3213460	rs476391	5'UTR	18090153	G/A
**11**	*MMP-1*	rs514921	rs644552	Promoter	18089805	A/G
**12**	*MMP-1*	rs498186	rs651159	Promoter	18089390	A/C
**13**	*MMP-1*	rs1799750	rs662028	Promoter	18088538:18088539	-/G
**14**	*MMP-12*	rs652438		Exonic (Asn357Ser)	18022346	A/G
				Intronic	18024778	T/A
				Intronic	18023958	G/C
				Intronic	18023848	G/A
				Intronic	18022569	A/G
				Intronic	18018358	A/G
**15**	*MMP-12*	rs632009	rs7123600	Intronic	18020490	C/T
			rs10895367	Intronic	18017018	A/G
**16**	*MMP-12*	rs11225442		Intronic	18019669	G/A
				Intronic	18013855	G/C
**17**	*MMP-12*	rs28381675	rs28381684	Intronic	18018010	A/G
**18**	*MMP-12*	rs2276109	rs17368582	Upstream	18013194	A/G
			rs17368659	Intronic	18021796	T/A
				Exonic	18020914	T/C
				Intronic	18016225	G/T
**19**	*MMP-9*	rs3918262	rs3787268	Intronic	7706054	A/G
				Intronic	7704015	G/A
**20**	*MMP-9*	rs13969	rs20544	Exonic	7705117	C/A
**21**	*MMP-9*	rs2250889	rs3918251	Exonic (Pro574Arg)	7704690	C/G
**22**	*MMP-9*	rs3918256	rs3918253	Intronic	7703243	A/G
			rs8113877	3' UTR	7707164	C/T
				Intronic	7701053	A/G
				Intronic	7701795	T/C
				Upstream	7697335	T/G
**23**	*MMP-9*	rs17576	rs3918249	Exonic (Gln279Arg)	7702509	A/G
			rs3918240	Intronic	7700408	C/T
			rs6965913	Upstream	7697906	C/T
				Upstream	7697393	C/T
**24**	*MMP-9*	rs2274755	rs3918242	Intronic	7701976	G/T
			rs13925	Promoter	7968266	C/T
			rs3918261	Exonic	7707119	G/A
			rs17577	Intronic	7705876	A/G
				Exonic (Arg668Gln)	7705395	G/A
			rs2236416	Intronic	7702859	A/G
			rs3918241	Upstream	7698025	T/A
**25**	*MMP-9*	rs3918278		Upstream	7697944	G/A
**26**	*MMP-9*	rs11697325		Upstream	7691635	A/G

SNPs are shown in 3' to 5' order. Contig Position numbering is relative to HuRef NCBI build 36.3. SNPs 6 and 13 (rs numbers 17884110 and 1799750 respectively) represent an insertion/deletion polymorphism. Amino acid changes are shown where present.

#### MMP-1

A panel of 21 validated SNPs was identified within the gene and its promoter region. However, rs488178 was found to be unsuitable for design and was excluded. Consideration of the linkage disequilibrium between the SNPs resulted in a final panel of 13 SNPs being genotyped, giving 87% coverage of validated SNP variation in *MMP-1*.

#### MMP-9

A panel of 23 validated SNPs was identified within the gene and its promoter region from the databases, which covered all known validated SNPs within the region. However, rs25650 was found to be non-polymorphic in the study cohort and was excluded from further analysis. Consideration of the linkage disequilibrium between the SNPs resulted in a final panel of 8 tagged SNPs being genotyped, giving 96% coverage of validated SNP variation in *MMP-9*.

#### MMP-12

A panel of 17 validated SNPs was identified within the gene and its promoter region, a selection which covered all known validated SNPs within the region. However, rs28360355, rs28381675 and rs28360356 were found to be unsuitable for assay design and were excluded. Consideration of the linkage disequilibrium between the SNPs resulted in a final panel of five SNPs being genotyped, giving 82% coverage of validated SNP variation in *MMP-12*.

### Genotyping of Study Samples

Full genotyping information was available in 977 COPD cases and 876 controls. Genotyping was carried out commercially by K- Bioscience using KASPar, an in-house validated competitive allele-specific polymerase chain reaction SNP genotyping system which utilises fluorescence resonance energy transfer quencher cassette oligonucleotides. Taqman, a rapid fluorophore based real- time PCR method, was used to genotype SNPs found to be unsuitable for KASPar assay design. As a quality control measure, approximately 5% of samples were genotyped in duplicate to check for concordance. There was 100% concordance between the duplicates, satisfying criteria for the assays to be accepted for further analysis.

### Statistical Analysis

**Figure 1:**
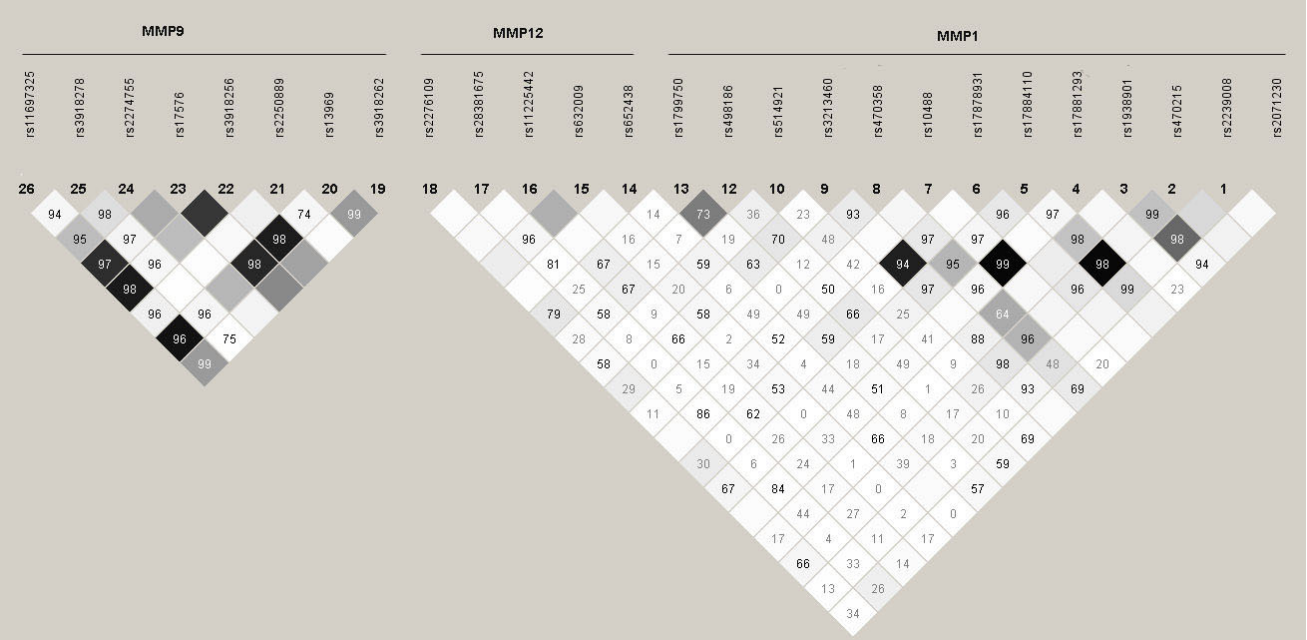
**LD plot in controls of SNPs in MMPs- 1, 9 and 12**. Estimated as r^2 ^using Haploview 4.1 output. SNP codes are provided in order of location along each gene; dark grey squares depict strong LD (1.0) with strong confidence, pale grey and white regions represent low LD, and the r^2 ^value is provided within each box. SNP positions are demonstrated 5' to 3' relative to contig postion (HuRef NCBI build 36.3).

Each of the SNPs in the three genes was analyzed for Hardy-Weinberg equilibrium (HWE) using PROC ALLELE in SAS/Genetics Release 9.1.3 [23], an empirical p-value of ≤ 0.01 was used as a cut-off, to reduce the likelihood of reporting false positives. To examine linkage disequilibrium, the correlation coefficient between SNP pairs within each gene in cases and controls was calculated using Haploview 4.1 [24] a software package that computes linkage disequilibrium and haplotype blocks from genotype data (figure 1).

Analyses of allele and genotype frequencies for the three *MMP *genes were performed using PROC ALLELE in SAS/Genetics. The adjusted p-values for genotype and allele frequencies were obtained using PROC LOGISTIC in SAS, adjusting for age, sex, smoking and centre.

As haplotypes of *MMP*s have been reported to be associated with COPD or decline in lung function [13,25], using FAMHAP18 [26] we examined haplotypes in all possible SNP combinations based on the eight SNPs in the *MMP-9 *gene on chromosome 20 and also in all combinations consisting of four or fewer SNPs from the 16 analysable SNPs (see below) in *MMP-1 *and *MMP*-*12 *combined on Chromosome 11. We considered only up to four haplotypes because of the optimal power considerations in FAMHAP18. We compared haplotype distributions for all cases versus controls, for GOLD severity III and IV cases versus controls and for GOLD severity IV only versus controls. We used as a cut off criterion a p < 0.01 for a significance test on the contingency table of all possible simulated haplotypes within possible SNP combination in cases and controls.

P-values were based on 10,000 simulations and since for this sample size the 95% confidence limits for 0.01 are +/- 0.002, we report all comparisons with a p-value less than 0.012. To allow for multiple testing we used the maximum estimated q-value that corresponds to p-values ≤ 0.012 with a high stringency estimate of the false discovery rate (FDR) [27,28] (using the q-value Package in R [29]).

Odds ratios for haplotype and individual SNP allele distributions are relative to the common haplotype or allele. The adjusted odds ratios with 95% confidence intervals, adjusting for age, sex, smoking and centre are estimated for haplotypes by weighted logistic regression as described previously [19] (PROC LOGISTIC in SAS), and for alleles by standard logistic regression.

Using PROC GLM in SAS [23] the quantitative trait associations between SNPs or haplotypes and the phenotype FEV_1 _were tested by multivariate regression using a similar weighted method to that used in logistic regression [19], adjusting for age, sex, smoking and centre. The adjusted means were determined using the LSMEANS option.

## Results

### Quality Control

The full list of SNPs analysed are shown in Table 4. The overall genotype call rate was 95% (range, 92-96%), and the accuracy was 100% according to duplicate genotyping of > 5% of samples. The use of high stringency cut-offs resulted in the loss of some genotyping data and reduction in the number of patients studied from the original sample set. Significant deviation from HWE was observed in controls for two *MMP-1 *SNPs, rs470358 and rs2071230. The assays for these two SNPs were sub-optimal, with overlap in the signals observed for homozygotes and hetrozygotes.

### Association Analysis

Table 5 compares the genotype frequencies between all cases and controls for the 26 SNPs analysed. There were no significant differences at p < 0.01 in either the crude analysis or the analysis adjusted for age, sex, smoking levels and centre. To evaluate potential genetic contribution to more severe manifestations of the disease, those with moderate levels of disease were excluded from the cases and patients with either severe or very severe disease (GOLD groups III and IV) were compared to controls. This comparison was also performed for cases with very severe disease only (GOLD group IV). This approach enabled evaluation of the potential underlying genetic contribution to disease severity phenotype within the study population with least reduction in power. While unadjusted analysis indicated significant associations between genotype frequencies of SNP 18 and more severe disease, these became non-significant with p > 0.01 when adjusted for age, sex, smoking and centre (Table 5). Table 6 analyses allele relationships for all SNPs.

**Table 5:** *MMP*s -*1, 12 and 9 *- Genotype frequencies in Controls and Cases

Gene	**SNP No**.	Locus	Genotypes	Controls	COPD all cases	Unadjusted P-value	Adjusted P-value**
***MMP*1**	**1**	**rs2071230**	AA/AG/GG	0.85/0.13/0.02	0.85/0.14/0.00	0.01	0.16
	**2**	**rs2239008**	GG/GA/AA	0.64/0.33/0.03	0.64/0.31/0.05	0.26	0.28
	**3**	**rs470215**	AA/AG/GG	0.39/0.47/0.14	0.40/0.47/0.13	0.61	0.86
	**4**	**rs1938901**	CC/CT/TT	0.51/0.41/0.08	0.48/0.42/0.10	0.25	0.22
	**5**	**rs17881293**	AA/AG/GG	0.89/0.10/0.01	0.89/0.11/0.00	0.65	0.74
	**6**	**rs17884110**	C/C/C	0.39/0.47/0.14	0.41/0.47/0.12	0.42	0.78
	**7**	**rs17878931**	AA/AC/CC	0.01/0.25/0.74	0.02/0.25/0.73	0.66	0.33
	**8**	**rs10488**	GG/GA/AA	0.89/0.11/0.01	0.88/0.11/0.00	0.6	0.75
	**9**	**rs470358**	TT/TC/CC	0.14/0.40/0.46	0.17/0.37/0.46	0.32	0.54
	**10**	**rs3213460**	GG/GA/AA	0.73/0.25/0.02	0.72/0.26/0.02	0.84	0.43
	**11**	**rs514921**	AA/AG/GG	0.50/0.42/0.08	0.50/0.42/0.08	0.99	0.94
	**12**	**rs498186**	AA/AC/CC	0.28/0.52/0.20	0.29/0.48/0.23	0.13	0.39
	**13**	**rs1799750**	G/G/G	0.26/0.52/0.22	0.28/0.47/0.25	0.13	0.45
***MMP*12**	**14**	**rs652438**	AA/AG/GG	0.90/0.10/0.00	0.90/0.09/0.00	0.34	0.87
	**15**	**rs632009**	CC/CT/TT	0.45/0.44/0.11	0.46/0.42/0.12	0.53	0.34
	**16**	**rs11225442**	GG/GA/AA	0.75/0.23/0.02	0.76/0.22/0.02	0.8	0.79
	**17**	**rs28381675**	AA/AG/GG	0.88/0.11/0.00	0.87/0.12/0.01	0.5	0.22
	**18**	**rs2276109**	AA/AG/GG	0.75/0.24/0.01	0.80/0.19/0.02	0.02	0.12
***MMP*9**	**19**	**rs3918262**	AA/AG/GG	0.66/0.31/0.03	0.63/0.32/0.05	0.12	0.15
	**20**	**rs13969**	CC/CA/AA	0.38/0.47/0.15	0.37/0.47/0.16	0.79	0.83
	**21**	**rs2250889**	CC/CG/GG	0.93/0.07/0.01	0.93/0.07/0.00	0.45	0.68
	**22**	**rs3918256**	AA/AG/GG	0.36/0.47/0.17	0.35/0.48/0.17	0.83	0.64
	**23**	**rs17576**	AA/AG/GG	0.43/0.45/0.12	0.42/0.45/0.13	0.62	0.58
	**24**	**rs2274755**	GG/GT/TT	0.71/0.26/0.02	0.73/0.25/0.02	0.69	0.79
	**25**	**rs3918278**	GG/GA/AA	0.96/0.04/0.00	0.95/0.05/0.00	0.46	0.13
	**26**	**rs11697325**	AA/AG/GG	0.39/0.46/0.15	0.37/0.47/0.16	0.64	0.64

**Gene**	**SNP No**.	**Locus**	**Genotypes**	**Controls**	**COPD** Severity IV*	**Unadjusted** **P-value**	Adjusted P-value**
***MMP12***	**18**	**rs2276109**	AA/AG/GG	0.75/0.24/0.01	0.83/0.15/0.02	0.008	0.03

**Gene**	**SNP No**.	**Locus**	**Genotypes**	**Controls**	**COPD** **Severity** III and IV*	**Unadjusted** **P-value**	Adjusted P-value**
***MMP12***	**18**	**rs2276109**	AA/AG/GG	0.75/0.24/0.01	0.81/0.17/0.01	0.007	0.08

*For GOLD classification severity III and IV, and severity IV, only SNPs with an unadjusted significant (p-value ≤ 0.01) genotypes difference between cases and controls are reported.

** P-values adjusted by logistic regression for age, sex, smoking and centre.

**Table 6:** *MMP*s -*1, 12 and 9 *- Allelic frequencies in Controls and Cases

Gene	**SNP No**.	Locus	Major/Minor Alleles	Minor Allele Frequencies		Unadjusted P-value	Adjusted P-value*	Adjusted Odds Ratio**
				Controls	COPD all cases			
***MMP*1**	**1**	**rs2071230**	A/G	0.08	0.07	0.4354	0.7453	1.045
	**2**	**rs2239008**	G/A	0.20	0.20	0.6539	0.5193	0.943
	**3**	**rs470215**	A/G	0.38	0.36	0.3502	0.5923	1.041
	**4**	**rs1938901**	C/T	0.28	0.31	0.1248	0.2060	0.904
	**5**	**rs17881293**	A/G	0.06	0.06	0.9887	0.8005	1.039
	**6**	**rs17884110**	-/C	0.38	0.36	0.2574	0.5656	1.044
	**7**	**rs17878931**	C/A	0.14	0.14	0.5514	0.4245	0.919
	**8**	**rs10488**	G/A	0.06	0.06	0.9190	0.7863	1.042
	**9**	**rs470358**	C/T	0.34	0.35	0.4928	0.6550	0.966
	**10**	**rs3213460**	G/A	0.14	0.15	0.5609	0.2504	0.889
	**11**	**rs514921**	A/G	0.29	0.29	0.9823	0.7495	1.026
	**12**	**rs498186**	A/C	0.46	0.47	0.4443	0.3265	0.931
	**13**	**rs1799750**	-/G	0.48	0.48	0.8539	0.6630	0.969
***MMP*12**	**14**	**rs652438**	A/G	0.05	0.05	0.4773	0.6428	1.078
	**15**	**rs632009**	C/T	0.33	0.33	0.9704	0.4266	1.063
	**16**	**rs11225442**	G/A	0.13	0.13	0.6509	0.6242	1.053
	**17**	**rs28381675**	A/G	0.06	0.07	0.3088	0.0910	0.784
	**18**	**rs2276109**	A/G	0.13	0.11	0.0659	0.3243	1.115
***MMP*9**	**19**	**rs3918262**	A/G	0.19	0.21	0.0591	0.0647	0.846
	**20**	**rs13969**	C/A	0.38	0.39	0.5351	0.9600	0.996
	**21**	**rs2250889**	C/G	0.04	0.04	0.4558	0.3645	1.185
	**22**	**rs3918256**	A/G	0.40	0.41	0.5548	0.9810	1.002
	**23**	**rs17576**	A/G	0.34	0.36	0.3622	0.4367	0.943
	**24**	**rs2274755**	G/T	0.16	0.14	0.3908	0.4962	1.072
	**25**	**rs3918278**	G/A	0.02	0.02	0.6433	0.0371	0.604
	**26**	**rs11697325**	A/G	0.38	0.39	0.3478	0.5696	0.959

*p-values adjusted by logistic regression for age, sex, smoking and centre.

** Odds ratios are relative to the major allele.

As described in the Methods, we then examined all possible haplotypes consisting of between two and four SNPs in the same chromosome for differences between the case groups defined above and controls. Adjusting for multiple testing the only significant differences in haplotype distributions were found when severe/very severe (GOLD groups III and IV) cases were involved. Table 7 shows the seven significant haplotypes together with their unadjusted p-values. Since the haplotype comprising SNPs 14 and 18 (p = 0.0086) is a subset of all the other significant combinations identified, we deduce that the significant results are a consequence of the association of SNPs 14 and 18 with disease severity in COPD, despite the apparently high false discovery rate of 0.54. The low LD (r^2 ^= 0.083) in controls between these two SNPs indicates the separate involvement of both SNPs in this association.

**Table 7:** *MMP - 1 and 12 *Risk Haplotypes related to disease GOLD severity III and IV combined versus controls

	SNP No							P-value^+^
		
Haplotypes	6	10	12	13	14	16	18	
**2-SNP**					x		x	0.0086
**3-SNP**		x			x		x	0.0116
**3-SNP**				x	x		x	0.0094
**3-SNP**					x	x	x	0.0076
**4-SNP**	x			x	x		x	0.0110
**4-SNP**		x		x	x		x	0.0054
**4-SNP**			x		x	x	x	0.0092

^+ ^P-values are by Monte-Carlo simulation on 10,000 runs.

Table 8 shows the actual haplotypes of SNPs 14 and 18 in the *MMP-12 *gene and their frequencies in severe/very severe disease (GOLD Stages III and IV) compared to controls. For completeness the table also shows the relationship of the alleles of each SNP alone with severity. Persons with the rare G allele of either SNPs 14 or 18 were protected against severe/very severe COPD. Compared with the common A-A haplotype of SNPs 14 and 18, subjects with the G allele at either locus had a significantly reduced risk of having severe/very severe COPD. Of note, and of importance from a population perspective, is that, based on the distribution in controls, 18% of the European population have at least one of these protective haplotypes, with an average risk 0.76 (95% CI: 0.61 to 0.94) times less than those with the common haplotype.

**Table 8:** *MMP*- *12 *haplotypes significantly different between GOLD Classification III and IV cases and controls

			**Frequency (%)**				
		**GOLD** **III and IV**	**Controls**	**p-value+**	Odds Ratio*		
		**(n = 641)**	**(n = 876)**		**Unadjusted**	Adjusted**	**95% CI**
	
**SNP 14** **(rs652438)**	**A**	96.2	94.7	0.0536	1		
	**G**	3.8	5.3		0.71	0.76	0.52 to 1.12
							
**SNP 18** **(rs2276109)**	**A**	90.0	87.0	0.0112	1		
	**G**	10.0	13.0		0.74	0.79	0.61 to 1.02
							
**Haplotypes of SNP 14 and SNP 18**	**A-A**	86.3	82.0	0.0086	1	1	
	**A-G**	9.8	12.7		0.73	0.78	0.60 to 1.00
	**G-A**	3.8	5.1		0.70	0.74	0.50 to 1.09
	**G-G**	0.1	0.2		0.36	0.96	0.09 to 10.63
							
	**A-A**	86.3	82.0	0.0039^++^	1		
	**A-G, G-A or G-G**	13.7	18.0		0.72	0.76	0.61 to 0.94

^+ ^P-values are by Monte-Carlo simulation on 10,000 runs.

^++ ^Adjusted for choice of the most significant 2 × 2 table with collapsed data.

* Odds ratios are relative to the common allele or haplotype.

** Adjusted for age, sex, smoking and centre.

Confining the analysis to cases only we examined the relationship between variation in *MMP-12 *SNPs 14 and 18 and their haplotypes to severity of disease using FEV_1 _as a severity measure. Weighted multivariate regression models, adjusting for age, sex, smoking and centre were used to test for the associations. Those individuals with the haplotype A-A had a significantly lower predicted FEV_1 _(42.62% versus 44.79%, p = 0.0129) when compared to patients with any haplotypes containing a G allele. We did not find a significant association between FEV_1 _and the alleles in SNP 14 and SNP 18 singly.

## Discussion

A number of studies have reported on the role of genetic variants of *MMP*s in genetic susceptibility to COPD or related phenotypes. However, the majority of studies have concentrated on the three well known promoter polymorphisms of these genes - *MMP*1-1607-/G (rs1799750), *MMP*9 -1562 C/T (rs3918242) and the *MMP*12-82A/G (rs2276109) in comparatively smaller sample numbers (Table 1).

Our study reports on the largest and most extensive screen of SNPs within the *MMP*s- 1, 9 and 12 genes undertaken to date and include the three promoter SNPs or SNPs that are in LD with the promoter SNPs which have been previously reported, in a larger sample size. All SNPs evaluated in the final analysis were in HWE in controls, had high call rates and concordant duplicates by genotyping, suggesting that this study was not likely to be influenced by genotyping errors. All subjects were of European descent and any potential effects of population stratification were minimised by recruiting patients and controls from each centre and confirmed by the fact that SNP genotype frequencies in the controls for each centre were found to be similar. Given the greater level of power in our study it is unlikely that effects of the magnitude observed in previous, less powered studies, have been missed. However, there may be subtle differences in phenotypes and association with quantitative traits such as lung function decline in the previous studies that we were not able to test. This is in part due to multiple testing issues, investigating numerous phenotypes could lead to false positives. Also, phenotype data for some clinical endpoints such as CT scans for emphysema has not been collected for our population. Although, this may cause difficulties with planning functional work, future work testing associated SNP's in other COPD cohorts characterised for specific phenotypes will address this.

The patients with very severe disease had a mean age that was less than the severe and moderate groups and this may reflect a survival bias.

We found that *MMP's *- *1 *and *9 *were not associated with COPD in our populations. This is an interesting result as these genes have previously been implicated in COPD by both rodent and a range of human studies. In the previous case control association studies, many of the associations in MMP's 1 and 9 have been demonstrated in non - Caucasian populations. Further to this, some of the studies use different phenotypic end points.

For the *MMP*-12 gene, we identified haplotypes associated with two SNPs, SNP 14 (rs652438) and SNP 18 (rs2276109), which showed associations with severe/very severe COPD. The associations with GOLD Stage III and IV disease suggest that these SNPs may play a modifier role in disease severity. We also showed that haplotypes of the two SNPs were significantly related to severity within the COPD cases, as determined by the quantitative levels of FEV1 within the cases. Those with the haplotypes A-G, G-A or G-G had higher FEV1 levels. It is of note that, evaluation of the association of SNP 18 (rs2276109) with COPD has been previously examined, but no effect was seen [13,30]. This probably reflects underpowered studies and the need to examine disease severity. SNP 14 (rs652438) has been previously shown to be associated with smoking induced COPD in a Chinese cohort [30], as well as being associated with a decline in lung function when considered as a haplotype with the *MMP*-*1 *promoter SNP 13 (rs1799750) [13]. The recent genome wide association study (GWAS) study in COPD by Pillai *et al *[31] didn't report association for *MMP-12 *in COPD even though there are SNPs in full linkage disequilibrium with rs2276109 and rs652438 on the illumina 550k platform used. However, this does not preclude the SNP's involvement in disease as they may have significance but not to genome wide level, access to the primary data would be of interest regarding this. Furthermore, in our population *MMP-12 *SNPs are associated with severe forms of COPD after haplotypic association, unlike the GWAS which is total COPD cases versus control population using single SNP association.

Although we noted a trend (p = 0.054) for association of SNP 14 (rs652438) with disease, our strict cut-off for significance indicates that it may require a much larger sample size to be confident of such an association. It is of note, however, that SNP 14 (rs652438) is a constituent of all the haplotypes found to be associated with severe/very severe disease, suggesting that it contributes to disease severity in COPD. It is also possible that this locus, or genetic variants in LD with rs652438, may affect susceptibility to COPD in the Chinese population. Whilst we did observe three haplotypes involving SNPs 13, 14 and 18 which were associated with severe/very severe disease (Table 7), there was no significant association for a 2-SNP haplotype with SNPs 13 and 14 only.

SNP 18 (rs2276109) with the alleles A/G is a known functional variant where the A allele shows a higher affinity for the transcription factor activator protein-1 (AP-1), resulting in increased expression in gene reporter assays. In the current study, the A-A haplotype composed of SNP 18 (rs2276109) and SNP 14 (rs652438) is over-represented in the cases, suggesting that increased *MMP*-*12 *levels may contribute to COPD pathogenesis. This is consistent with observations of increased *MMP*-*12 *activity in the lungs of patients with COPD and with observations in a rodent model of disease where *MMP-12 *knock-outs are protected against smoking-induced emphysema.

A recent paper by McAloon *et al *[32], screened *MMPs 1, 3 *and *12 *relatively comprehensively in AATD with associations being found with gas transfer. This could allude to *MMP - 12's *role in parenchymal disease pathophysiology rather than airways disease, especially when considered with association in this study against severe forms of COPD and in previous studies with emphysema.

## Conclusions

While this study provides suggestive evidence for a contribution of genetic variation in *MMP-12 *to disease severity in COPD, we do not find evidence for the involvement of *MMP*s- *1 and 9 *in COPD. This merits further investigation of *MMP-12 *associations in similar or larger cohorts.

## Competing interests

The authors declare that they have no competing interests.

## Authors' contributions

The authors IH, SC, JL, LD, COC and NK made substantial contribution to the conception and design of the study, and analysis and interpretation of the data. Whilst SJ, KM, TG, JR, RR, AM, SD, VK, WM, JS, PH, MM and SM made a substantial contribution to the collection of the resource and an intellectual contribution to the study design.

## Pre-publication history

The pre-publication history for this paper can be accessed here:

http://www.biomedcentral.com/1471-2350/11/7/prepub
